# A conversation with Dr. Paul Marasco, PhD, Laboratory for Bionic Integration, Department of Biomedical Engineering, Lerner Research Institute, Cleveland Clinic

**DOI:** 10.1017/cts.2022.528

**Published:** 2023-02-28

**Authors:** Kathy Siranosian

**Affiliations:** Clinical Research Forum, Washington, DC, USA

## Top 10 Clinical Research Achievement Awards Q & A

This article is part of a series of interviews with recipients of Clinical Research Forum’s Top 10 Clinical Research Achievement Awards. This article is with Dr Paul Marasco, PhD, Laboratory for Bionic Integration, Department of Biomedical Engineering, Lerner Research Institute, Cleveland Clinic. Dr Marasco studies the sensory nervous system, with a specific focus on brain organization and human–machine cooperation. Dr Marasco received a 2022 Top 10 Clinical Research Achievement Award for *Neurorobotic fusion of prosthetic touch, kinesthesia, and movement in bionic upper limbs promotes intrinsic brain behaviors* [1]. *The interview has been edited for length and clarity.*

## How did you get started in clinical research?

I took a nontraditional path. Ever since I was a kid, I wanted to get involved with building prosthetics for people but growing up in a small town in rural Colorado, there weren't a lot of options to help me reach that goal. Before getting into science I was a service manager at a bike shop. It was a fairly big operation, with about $2 million in business a year, and working there helped me meet a lot of different people, including doctors. Those connections led me to a job in a developmental molecular genetics lab. Up until then, I had always felt like a square peg in a round hole. But in that research setting, it was different. People recognized me and liked what I was doing and eventually, with their encouragement, I applied to and was accepted into a neuroscience program.

## Is that where you got interested in sensory systems?

Yes, at Vanderbilt University I worked in Ken Catania’s lab, which is focused on investigating mammalian sensory systems using unusual animal models like star-nosed moles and electric eels. Through that work I got connected with another group that was looking for people to study a sense of touch for prosthetic limbs in amputees. I was able to take what I had learned about sensory systems and the different animal models and consolidate it into studying these really interesting human situations where people have had their nerves redirected. Everything I'd done across the decades started fitting together and helped me begin doing the kind of science I do, which is cross-cutting and brings in a lot of different perspectives. My research is focused on building prosthetic limbs but it’s based on understanding how the brain processes sensory motor information and interacts with these different devices.

## How has the field changed in the past few years?

Prosthetics are becoming increasingly sophisticated, and we are now using advanced bionic approaches with surgical, implanted, and surface signal detection strategies that can access the intentional motor control signals directly from the brain and nerves. The research we do is mostly with hands and arms, which turns out to be a really interesting engineering problem because hands and arms are elegant systems that actually work autonomously. We like to think that we’re in control, that it’s the brain saying, “Hey, go get that—go grab my coffee,” and then the arm goes and does it. The sensation that we get back tells us that what happened was anticipated and helps us believe it was in our control. But in our award-winning paper we looked at how bionic limbs can communicate with the people who are wearing them and we start to explore how innate and reflexive these actions really are.

## What were the key findings?

The paper included two significant breakthroughs. The first was the one I just described – we integrated functional improvements with reflexivity. We want people to start relating to a prosthetic reflexively without knowing it. We want their bodies working better without them even being aware of it, which is exactly how you work when you have your native arms. It’s this kinesthesia portion of the work that we do that’s really different. Kinesthesia is a sense that most people don’t even know they have, and what we’ve realized is that it’s very difficult to engage with. But if you check out the paper, you’ll see that we found that the more channels of natural communication that you have, the more human like the function of the limb. In two participants with proximal arm amputation, the neurorobotic fusion of touch, kinesthesia, and intuitive motor control promoted performance levels that were stratified toward able-bodied functional behaviors and away from standard-of-care prosthetic users.

## And what was the second breakthrough?

Our other key innovation – and in many ways, this was the most important one – was the metrics. We had to build the metrics to be able to show that the device is actually helping people return to normal function. Before this, we were using old tools that don’t even adequately describe the function of a basic prosthetic limb to characterize the function of an advanced neurally integrated system, which has caused a variety of challenges when dealing with insurance companies, doctors, and other stakeholders. So we built a suite of metrics that looks specifically at all of the different pieces associated with returning function to someone with an advanced prosthesis – and it’s not just based on timing. It’s based on behaviors, on reflexes, on all the innate pieces, like how the brain compensates for its own intrinsic error. When people ask me about what’s going to be the next big thing in the field, they tend to want to focus on the technology. But my answer is that it’s the metrics. Not having metrics was standing in the way of progress. With the metrics, we can show that people are using bionic prosthetics like natural limbs, in ways that should reduce the compensatory and sound-side overuse injuries that occur with traditional prostheses. They help us continue to improve, and they help us justify the use of advanced systems.

## What do you like most about being a clinical researcher?

What I love about my job is that every day is a new and exciting experience. As a clinical researcher, I follow wherever the data tells me to go. That can lead to totally unexpected places, which is where the breakthroughs happen. Exploring these different paths is very motivating, as is knowing that the scientific questions we’re trying to answer and the devices that we are building are all focused on addressing problems that people have. The clinical piece is so important. We’re clinically embedded in Cleveland Clinic’s Charles Shor Epilepsy Center and the Cleveland VA Medical Center’s amputee clinic, and we’re engaged with finding solutions that have real impacts on people’s lives.
